# Corneal Collagen Cross-Linking in Pellucid Marginal Degeneration: 2 Patients, 4 Eyes

**DOI:** 10.1155/2015/840687

**Published:** 2015-05-11

**Authors:** Serife Bayraktar, Zafer Cebeci, Merih Oray, Nilufer Alparslan

**Affiliations:** Department of Ophthalmology, Istanbul Faculty of Medicine, Istanbul University, 34093 Istanbul, Turkey

## Abstract

*Purpose*. To report the long-term results of corneal collagen cross-linking (CXL) with riboflavin and ultraviolet-A irradiation in 4 eyes of 2 patients affected by pellucid marginal degeneration (PMD). *Methods*. This study involved the retrospective analysis of 4 eyes of 2 patients with PMD that underwent CXL treatment. Of the eyes, three had only CXL treatment and one had CXL treatment after an intrastromal corneal ring segment implantation. We have pre- and postoperatively evaluated uncorrected distance visual acuity (UDVA), best corrected distance visual acuity (BCDVA), corneal topography (Pentacam), specular microscopy, and pachymetry. *Results*. Patient 1 was a woman, aged 35, and Patient 2 was a man, aged 33. The right eye of Patient 1 showed an improvement in her BCDVA, from 16/40 to 18/20 in 15 months, and her left eye improved from 12/20 to 18/20 in 20 months. Patient 2's right eye showed an improvement in his BCDVA, from 18/20 to 20/20 in 43 months, and his left eye improved from 16/20 to 18/20 in 22 months. No complications were recorded during or after the treatment. *Conclusion*. CXL is a safe tool for the management of PMD, and it can help to stop the progression of this disease.

## 1. Introduction

Pellucid marginal degeneration (PMD) is a rare, idiopathic, bilateral, progressive noninflammatory thinning corneal disorder. It is characterized by a peripheral band of thinning, usually occurring in the inferior quadrant in a crescentic fashion. A 1-2 mm margin of normal cornea lies between the thinning and the limbus [1, 2].

Patients present with a decrease of visual acuity in their thirties to fifties because of high and irregular astigmatism [3]. Nonsurgical approaches to the management of PMD include spectacles and contact lenses [4, 5]. Surgical options include intrastromal corneal rings, thermocauterization, and keratoplasty [6, 7].

Corneal collagen cross-linking (CXL) with riboflavin and ultraviolet-A (UVA) light is a corneal tissue strengthening technique, which uses riboflavin as a photosensitizer and UVA to increase the formation of intra- and interfibrillar covalent bonds by photosensitized oxidation [8, 9].

In this study we present the long-term results of corneal collagen CXL with riboflavin and UVA irradiation in the eyes of two patients affected by PMD.

## 2. Patients and Methods

After signing an informed consent form and receiving topical anesthetic riboflavin UVA-induced, a corneal collagen CXL was performed in the following stages: pilocarpine 2% drops for 30 minutes preoperatively; topical anesthesia with proparacaine HCl 0.5% drops (Alcaine, Alcon, Fort Worth, Texas, USA) before epithelial removal; corneal mechanical epithelial scraping of an area 9 mm in diameter; preirradiation riboflavin solution (Ricrolin; SOOFT Italia S.p.A., Montegiorgio, Italy) applied every 3 minutes for 30 minutes; exposure to a solid-state UVA illuminator (CBM, Vega CSO, Florence, Italy) for 30 minutes; and irradiation of a slightly inferiorly decentered area 9 mm in diameter and approximately 1 mm from the limbus (energy delivered at 3 mW/cm^2^). At the end of the treatment, a therapeutic bandage soft contact lenses were applied for one-week period, and an antibiotic regimen of moxifloxacin (Vigamox %0.5, Alcon, Fort Worth, Texas, USA) drops four times a day for 2 weeks and topical corticosteroid (dexamethasone, Dexa-sine Se 0.4 mL/1.3 mg, Liba, Istanbul, Turkey) drops twice a day for 3 weeks was administered.

### 2.1. Patient 1

**Figure 1:**
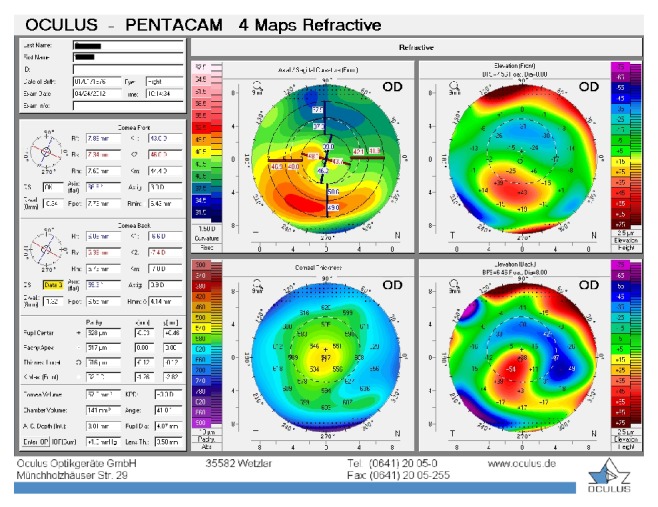
Patient 1, right eye before the treatment.

**Figure 2:**
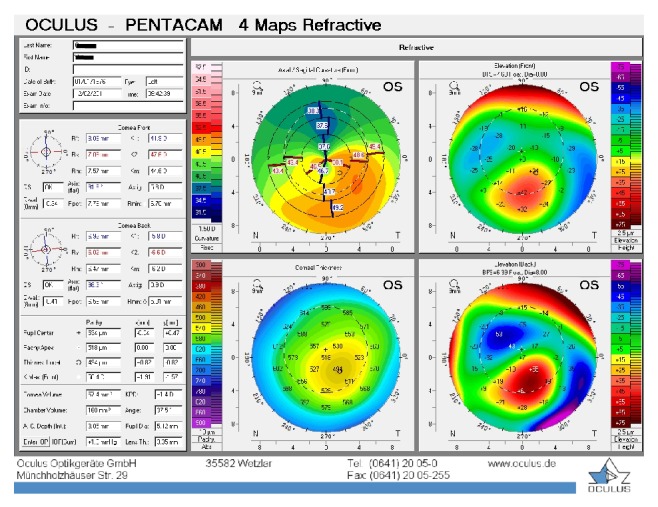
Patient 1, left eye before the treatment.

The first patient was a 35-year-old contact lens-intolerant woman with bilateral PMD who had reported a progressive impairment of vision for the past 6 years. In the right eye, UDVA and BCDVA were 12/40 and 16/40, respectively, with a correction of −1.00 −3.00 × 85. In the left eye, UDVA and BCDVA were 8/40 and 12/20, respectively, with a correction of +1.00 −4.00 × 105. A corneal topography obtained by Pentacam (Pentacam, OCULUS GmbH, Wetzlar, Germany) showed an irregular astigmatism with inferior corneal steepening (Figures 1 and 2). The thinnest corneal thickness was 486 *μ* in the right eye and 499 *μ* in the left eye. A noncontact endothelial specular microscopy (KONAN Medical Inc., Model NSP 9900, Hyogo, Japan) recorded endothelial cell densities of 2294 cells/mm^2^ in the right eye and 2545 cells/mm^2^ in the left eye. Intraocular pressure, evaluated by Goldmann applanation tonometry, was 12 mmHg in both eyes. The results of the fundus examination were normal in both eyes.

A riboflavin UVA-induced corneal collagen CXL was first performed in the left eye. After 4 months, an intrastromal corneal ring segment (Mediphacos, 5.0 mm, 160^0^, 300 *μ*, Belo Horizonte, Brazil) was inserted into an intrastromal pocket created by a femtosecond laser (IFS, Advanced Femtosecod Laser, AMO, Illinois, USA) in the right eye. A riboflavin UVA-induced corneal collagen CXL was performed in the same eye 6 weeks after implantation.

The treated eyes were examined 1 day, 1 week, 1 month, 3 months, and every 6 months after the treatments. No toxic effects or damage to the limbal region were observed during reepithelialization or during follow-ups. The UDVA and BCDVA were 8/40 and 10/20, respectively, with a correction of +1.00 −3.00 × 100 in the left eye over the first year. At the last visit, 20 months after treatment, the BCDVA had improved to 18/20, with a correction of +2.25 −4.75 × 90. In the right eye, the UDVA and BCDVA were 14/20 and 16/20, respectively, with a correction of +2.00 −3.50 × 85 over the first year. The BCDVA improved to 18/20, with a correction of −1.00 × 85 15 months after treatment.

**Figure 3:**
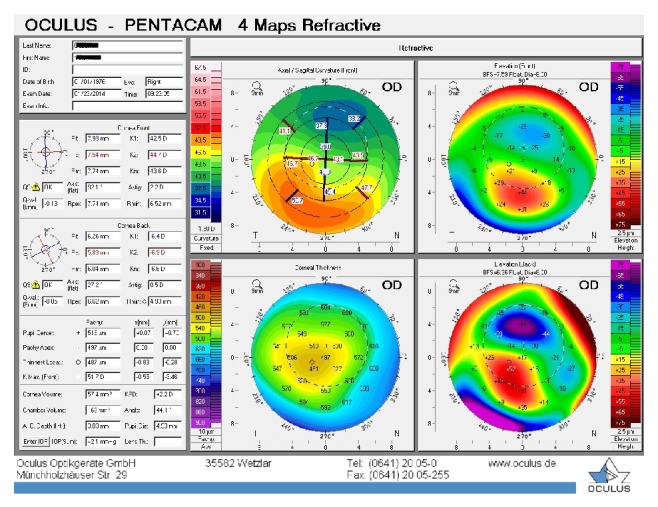
Patient 1, right eye at the last visit.

**Figure 4:**
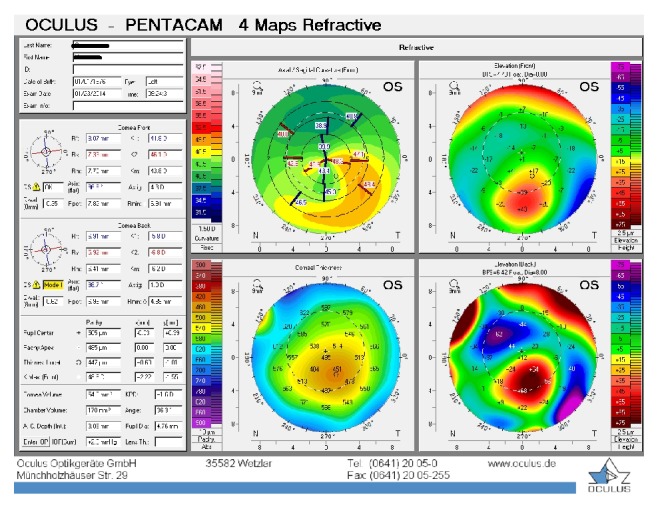
Patient 1, left eye at the last visit.

The baseline flattest meridian keratometry, the steepest meridian keratometry, and the apex of the ectasia power were 44.9 D, 49.5 D, and 55.0 D, respectively, in the right eye, and they were 42.1 D, 47.1 D, and 51.0 D, respectively, in the left eye. Twenty-two months after the first examination, the keratometry parameters were 42.2 D, 44.5 D, and 51.8, respectively, in the right eye and 41.4 D, 45.8 D, and 49.5 D in the left eye (Figures 3 and 4). A noncontact endothelial specular microscopy recorded endothelial cell densities of 2481 cells/mm^2^ in the right eye and 2587 cells/mm^2^ in the left eye at the last visit. The patient described an improved, comfortable quality of vision.

### 2.2. Patient 2

**Figure 5:**
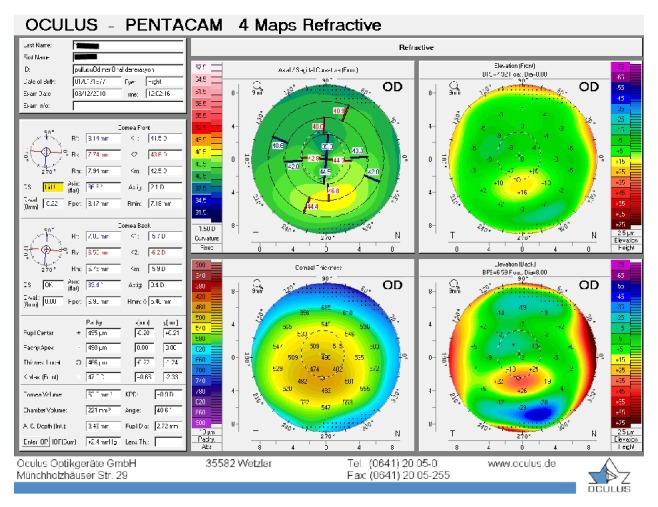
Patient 2, right eye before the treatment.

**Figure 6:**
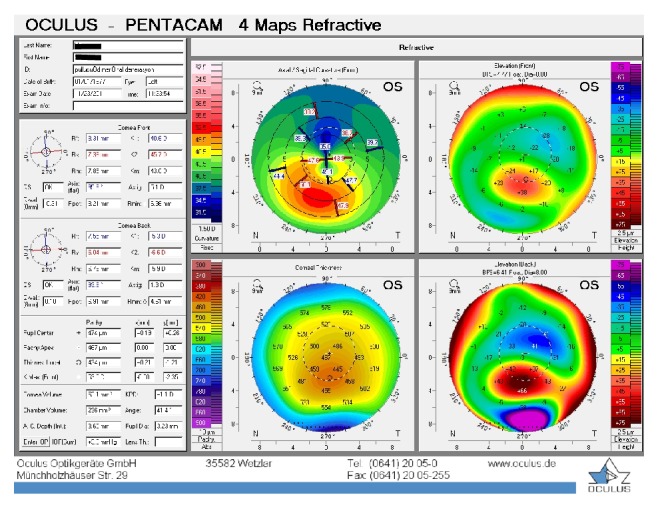
Patient 2, left eye before the treatment.

The second patient was a 33-year-old man with bilateral PMD who reported progressive impairment of vision and who had needed to change his eyeglasses every 3 months for the past 3 years. In the right eye, UDVA and BCDVA were 10/40 and 18/20, respectively, with a correction of −3.00 × 85. In the left eye, UDVA and BCDVA were 10/40 and 16/20, respectively, with a correction of −2.50 × 105. A corneal topography was obtained by Pentacam, and it showed irregular astigmatism with inferior corneal steepening (Figures 5 and 6). The thinnest corneal thickness was 466 *μ* in the right eye and 474 *μ* in the left eye. A noncontact endothelial specular microscopy recorded endothelial cell densities of 2445 cells/mm^2^ in the right eye and 2538 cells/mm^2^ in the left eye. The intraocular pressure, as evaluated by Goldmann applanation tonometry, was 13 mmHg in the right eye and 14 mmHg in the left eye. The results of a fundus examination were normal in both eyes. A riboflavin UVA-induced corneal collagen CXL was performed first in the right eye and then in the left eye 20 months later when the progression was seen.

The treated eyes were examined 1 day, 1 week, 1 month, 3 months, and every 6 months after the treatments. No toxic effects or damage to the limbal region were observed during reepithelialization or follow-ups. The UDVA and BCDVA were 8/40 and 20/20, respectively, with a correction of +0.50 −3.00 × 90 in the right eye over the first year. At the final visit, 43 months after the treatment, the BCDVA was 20/20 with a correction of +0.50 −2.50 × 90. In the left eye, the UDVA and BCDVA were 10/20 and 16/20, respectively, with a correction of +1.00 −5.00 × 90 over the first year. The BCDVA improved to 18/20, with a correction of +1.00 −5.00 × 90, 22 months after treatment.

**Figure 7:**
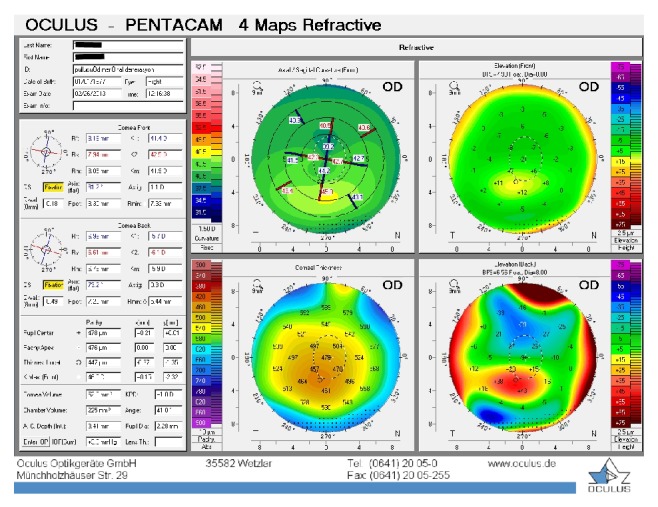
Patient 2, right eye at the last visit.

**Figure 8:**
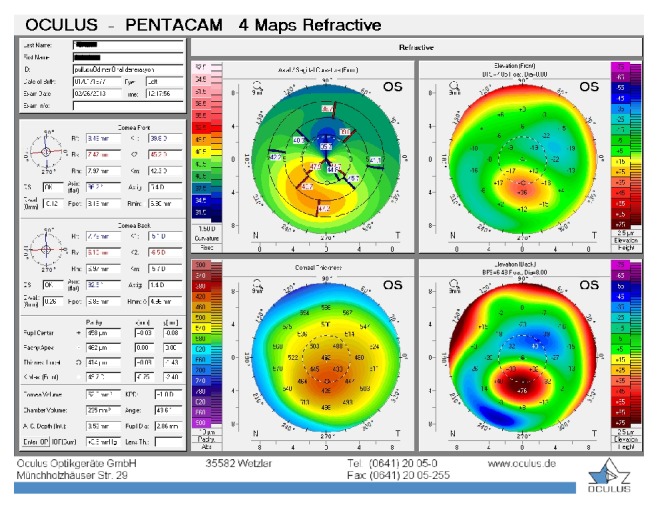
Patient 2, left eye at the last visit.

The baseline flattest meridian keratometry, the steepest meridian keratometry, and the apex of the ectasia power were 41.5 D, 43.6 D, and 47.0 D, respectively, in the right eye and 40.5 D, 43.2 D, and 46.2 D, respectively, in the left eye. The keratometry parameters were 40.9 D, 42.9 D, and 45.5, respectively, in the right eye and 40.8 D, 44.5 D, and 51.0 D, respectively, in the left eye 43 months after the first examination (Figures 7 and 8). A noncontact endothelial specular microscopy recorded endothelial cell densities of 2688 cells/mm^2^ in the right eye and 2475 cells/mm^2^ in the left eye at the last visit. The patient described a comfortable quality of vision.

## 3. Discussion

PMD is a typically bilateral, clear, inferior, and peripheral corneal thinning disorder. The cornea protrudes above the area of thinning, resulting in high and irregular astigmatism. The initial treatment of PMD can include optical correction and contact lenses. When the disease progresses to more advanced stages, surgical procedures such as thermocauterization, wedge resection, intracorneal ring segments, penetration, and lamellar keratoplasty may be necessary [2, 5].

Corneal collagen CXL has been used to treat progressive keratoconus since it was first introduced [9]. Nevertheless, new applications are under investigation and have shown promising results, such as the treatment of postoperative LASIK ectasia [10], the strengthening of recalcitrant corneal ulcerations [11], the stiffening of the peripapillary sclera for neuroprotection as a possible therapy for low-tension glaucoma [12], and bullous keratopathy [13].

The use of collagen CXL for keratoconus could be extended to inhibit the progression of corneal ectasia in PMD. Steppat et al. did not note any side effects and/or progression of the disease after an 18-month follow-up with eight PMD patients treated by CXL [14]. Additionally, Kymionis et al. performed simultaneous photorefractive keratectomy and CXL in a 34-year-old woman with PMD in both eyes [15]. A corneal topography revealed significant improvements in both eyes. Spadea reported the results of CXL in a 43-year-old patient with PMD [16]. In this case, the corrected distance visual acuity improved from 20/200 to 20/63 at 3 months, and it was stable through the 12-month interval.

In our patients, CXL led to prevent the progression of the disease. Also corneal flattening and a significant, stable improvement of UDVA and BCDVA were seen with no side effects during a time interval of 2 years. There was no loss in the corneal endothelial densities, which indicates that it is a safe procedure for managing PMD.
